# Skin and Scars Across Cultures: A Systematic Review on the Dermatological Impacts of Domestic Abuse

**DOI:** 10.7759/cureus.97145

**Published:** 2025-11-18

**Authors:** Rajah Darwish, Marina Zakhary, Kaleigh M Wingate, Shermeeka Hogans-Mathews

**Affiliations:** 1 Dermatology, Florida State University College of Medicine, Tallahassee, USA; 2 Psychiatry, Florida State University College of Medicine, Tallahassee , USA; 3 Psychiatry, Florida State University College of Medicine, Tallahassee, USA; 4 Family Medicine and Rural Health, Florida State University College of Medicine, Tallahassee, USA

**Keywords:** culture, dermatology, domestic abuse, intimate partner violence, women

## Abstract

Despite the prevalence of domestic abuse and its profound dermatological impacts, there remains a scarcity of comprehensive research exploring this issue through a cross-cultural lens. The objective of this review was to synthesize available evidence on the dermatological impacts of domestic abuse across cultural contexts. This systematic review was conducted and reported according to the PRISMA guidelines. Research studies focusing on individuals who have experienced domestic abuse in various cultural contexts were included. The primary exposure was domestic abuse, and studies investigating its dermatological impacts were eligible for inclusion. Eleven studies were included in the review. The majority of the included studies were observational studies (n=8) while four studies were case reports, covering diverse regions including North America, Europe, South America, Asia, and Australia. The majority of participants were female survivors of intimate partner violence (IPV), with mean ages typically falling in the early to mid-30s. The most frequently reported dermatological injuries included bruises and contusions primarily affecting the face, neck, upper and lower limbs, and scalp. Lacerations and incised wounds were reported in multiple studies, followed by abrasions, ecchymosis, burns, scarring, and permanent disfigurement. While the types of injuries were largely consistent across geographical settings, some regional patterns were observed. The findings highlight the global burden of dermatological injuries in survivors of domestic abuse. The findings suggest that while patterns of skin injuries are relatively consistent, cultural and regional factors may influence the nature and severity of injuries, warranting further investigation.

## Introduction and background

Domestic abuse is a widespread and devastating public health issue, affecting individuals of all genders, ages, and cultural backgrounds [1,2]. It includes a variety of abusive practices, such as economic control, psychological coercion, emotional manipulation, physical violence, and sexual abuse [3]. Domestic abuse has many repercussions, leaving victims with both visible and invisible consequences. Dermatological signs, including burns, scars, bruises, and other injuries, are among the most obvious and immediate physical effects [4]. Though their identification and interpretation might differ greatly among cultural contexts, these skin conditions frequently act as silent witnesses to the trauma that survivors have experienced [5,6].

The relationship between domestic abuse and dermatological health is deeply intertwined with cultural norms and societal attitudes. Dermatological conditions may go undiagnosed or be misdiagnosed in certain cultures due to the stigma associated with domestic abuse and traditional gender roles [7,8]. The stigma associated with domestic abuse may even prevent victims from seeking medical assistance in certain cultures [9]. Cultural barriers, lack of training, and lack of understanding of the subtle dermatological presentations of abuse can all make it difficult for healthcare practitioners to recognize and address these indicators. Cultural heterogeneity greatly impacts the ability of healthcare systems to offer survivors timely and adequate care [10-12]. Dermatological signs of abuse frequently carry a heavy psychological weight in addition to the physical effects [13,14].

Despite the prevalence of domestic abuse and its profound dermatological impacts, there remains a scarcity of comprehensive research exploring this issue through a cross-cultural lens. Existing literature tends to focus on isolated aspects of abuse or specific cultural settings, leaving a gap in understanding the global and culturally oriented dimensions of the problem. This systematic review aims to fill this gap by synthesizing evidence on the dermatological impacts of domestic abuse across cultures. It explores how cultural norms shape the recognition and management of dermatological manifestations caused by abuse. By providing a holistic understanding of the issue, this review seeks to inform healthcare providers, policymakers, and researchers, ultimately contributing to improved care and support for survivors of domestic abuse globally. The objective of this review was to synthesize available evidence on the dermatological impacts of domestic abuse across cultural contexts. The review question was: What are the dermatological manifestations of domestic abuse across cultures?

## Review

Material and methods

This systematic review was conducted and reported in accordance with the Preferred Reporting Items for Systematic Reviews and Meta-Analyses (PRISMA) guidelines [15,16].

Eligibility Criteria

Research studies focusing on individuals who have experienced domestic abuse in various cultural contexts were included. Domestic abuse was defined broadly to encompass physical and emotional. The primary exposure was domestic abuse, and studies investigating its dermatological impacts were eligible for inclusion. Only peer-reviewed published studies in the English language were included. There were no restrictions on the publication date. Editorials, commentaries, letters to the editors, and conference papers were excluded.

Information Sources and Search Strategy

An electronic search was conducted in the following databases: MEDLINE (Ovid), EMBASE, AMED, CINAHL, Cochrane Library, Web of Science, and Scopus. There were no time restrictions on the search dates. The searches were conducted from the earliest records to 7th February 2025. Additionally, a hand search of the reference lists of included studies was performed to identify potentially eligible studies. The search strategy combined keywords and Medical Subject Headings (MeSH) terms related to domestic abuse, dermatological impacts, and cultural contexts. Boolean operators (AND, OR) were used to refine searches.

Study Selection

The retrieved records from different databases and other sources were imported into Covidence [16]. Duplicates were removed, and the remaining articles were moved to title and abstract screening. Titles and abstracts were primarily used to screen the searched studies and identify full-text articles. Reviewers performed title and abstract screening, and any disagreements were resolved through consensus discussions. The articles with relevant titles and abstracts were moved to the full-text screening stage.

Full-text articles were retrieved after their selection from the screening process and further assessed for suitability. Full-text articles were also retrieved for those articles in which eligibility was uncertain based on the title and abstract alone. Reviewers performed full-text screening, and any disagreements were resolved through consensus discussions. The full text of the articles that fulfill the eligibility criteria was moved to the data extraction stage.

Data Collection Process and Data Items

An Excel sheet was designed and piloted for data extraction. Reviewers thoroughly studied the full text and extracted data from the articles that fulfilled the eligibility criteria. Any disagreements during data extraction were resolved through consensus discussions. Data from all included studies were systematically populated into the customized data extraction sheet. The sheet captured information regarding both study characteristics and exposure details. The primary outcome was the dermatological conditions (e.g., scars, burns, bruises, and other skin-related manifestations) caused by domestic abuse. The data items, including authors' names, year of publication, settings, country, study design, exposure-related details (such as abuse type and other relevant details), dermatological impacts, and cultural considerations, were extracted from the included studies.

Risk of Bias Assessment

The Newcastle-Ottawa Scale was used for risk of bias assessment of the observational studies, while the JBI critical appraisal checklist for case reports was used for risk of bias assessment of the case reports [15].

Synthesis of Results

The extracted data were synthesized and presented in comprehensive tables, accompanied by detailed explanations. The narrative synthesis (systematic review) was presented as per PRISMA guidelines.

Results

**Figure 1:**
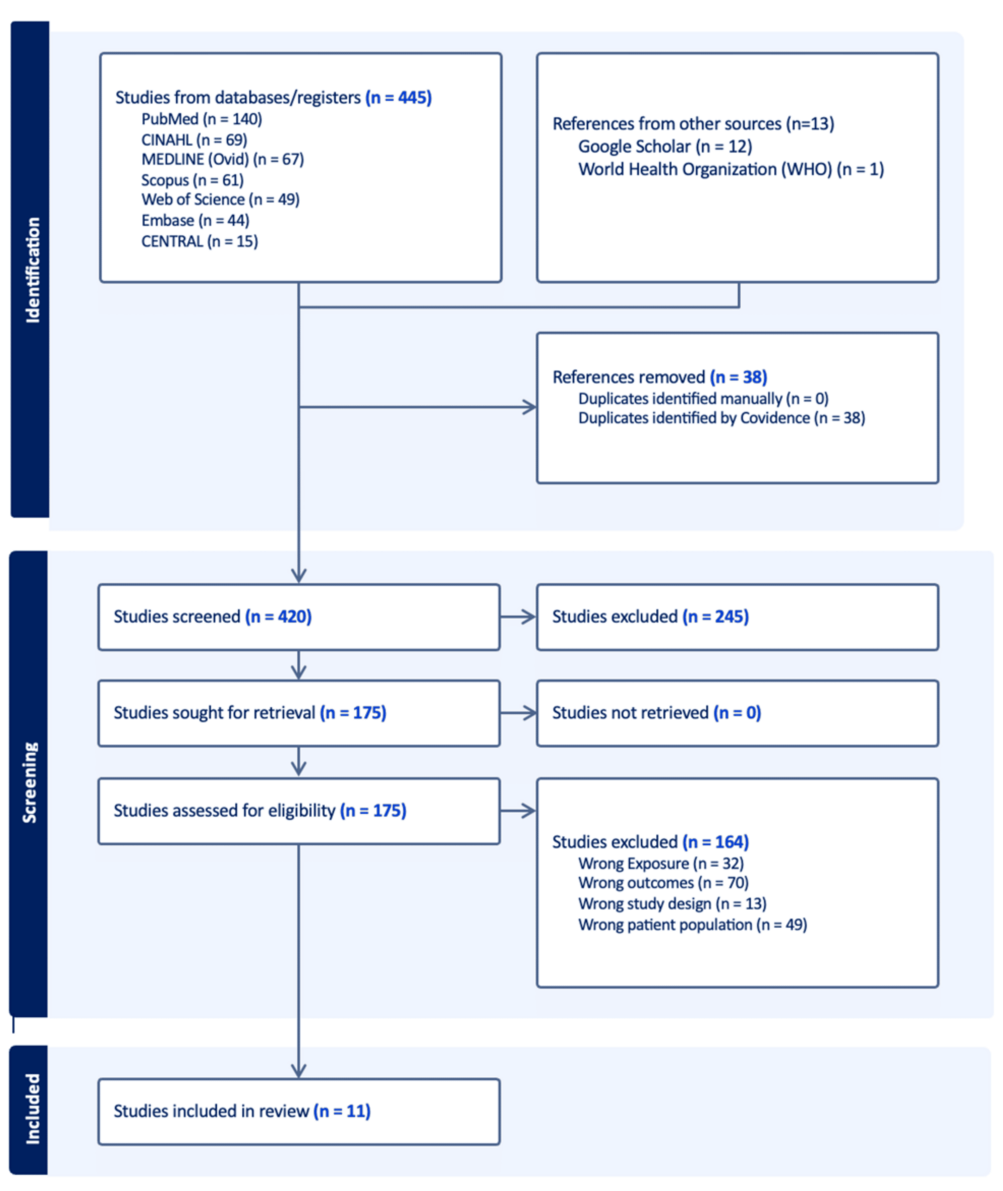
PRISMA flowchart showing study selection process PRISMA: Preferred Reporting Items for Systematic Reviews and Meta-Analyses.

Initial searching identified 458 records (445 from different electronic databases and 13 from other sources). Thirty-eight were duplicate records; thus, 420 remained for the title and abstract screening stage. At the title and abstract screening stage, 245 were excluded, while the full text of the remaining 175 studies was retrieved. A total of 164 full-text studies were excluded, and ultimately, 11 studies were included in the systematic review [7,11,17-25] (Figure 1).

The majority of the included studies were observational studies (n=8) [7,11,17-19,21,24], while four studies were case reports [20,22,23,25], covering diverse regions including North America, Europe, South America, Asia, and Australia. The sample sizes ranged from single-case reports to large-scale studies with up to 332 participants. The majority of participants were female survivors of intimate partner violence (IPV), with mean ages typically falling in the early to mid-30s. Ethnic distribution was variable, with some studies explicitly reporting ethnic backgrounds, while others did not provide this information. The most frequently reported dermatological injuries included bruises and contusions, lacerations and incised wounds, abrasions and ecchymosis, burns, and scarring and permanent disfigurement.

Bruises and contusions were reported in up to 96% of cases, primarily affecting the face, neck, upper and lower limbs, and scalp [11,17]. Lacerations and incised wounds were documented in multiple studies, often located on facial regions, lips, and extremities [18,22]. Abrasions and ecchymosis were found across different body regions, including the thighs, back, upper limbs, and periocular areas [20,25]. Burns ranged from cigarette burns to chemical burns and second-degree burns from fire. Burns were often associated with severe and permanent scarring, particularly in the face, neck, and torso [23,25]. Scarring and permanent disfigurement were highlighted as the long-term impact of abuse, with scars being frequently documented on the neck, hands, and limbs [21,25]. One case involved facial disfigurement due to chemical burns, leading to permanent disability [17].

While the types of injuries were largely consistent across geographical settings, some regional patterns were observed. North American Studies (Canada, USA) reported a high prevalence of facial and neck injuries, with bruising and lacerations being dominant [7,11]. European Studies (Portugal, Croatia, Turkey) showed perioral and soft tissue injuries as significant concerns, along with ligature marks and extensive bruising [19,20,25]. South American and Middle Eastern Studies (Brazil, Jordan) highlighted severe burns, extensive bruising, and soft tissue damage as frequent manifestations [17,21]. Asian Studies (Indonesia, Nepal) reported burn-related injuries and ocular trauma, including subconjunctival hemorrhage [18,23].

These findings highlight the global burden of dermatological injuries in survivors of domestic abuse. The findings suggest that while patterns of skin injuries are relatively consistent, cultural and regional factors may influence the nature and severity of injuries, warranting further investigation (Table 1). The risk of bias assessment of observational studies showed that the included observational studies had a score of 6 to 8 on the Newcastle-Ottawa Scale, demonstrating medium to high quality (Table 2). Similarly, risk of bias assessment of case reports showed that the included case report had a score of 6 to 7 on the JBI critical appraisal checklist for case reports, demonstrating medium to high quality (Table 3).

**Table 1:** Summary of the studies included

Author name (year of publication)	Country	Study Design	Participant Characteristics	Main findings (Dermatology Related)
Bhandari et al. (2006) [7]	Canada	Observational study design (quantitative)	263 female survivors of intimate partner violence, with a mean age of 33.4 ± 9.2 years, were included. Ethnicity Caucasian 62% African American 20% Latino 2% Native American 8% South Asian 0.5% Asian 3% South American 0.5% Mixed ethnicity 3%	22% women had skin (integumentary system) related injuries which included burns (2 occurrences), bruises (12 occurrences), scratches (3 occurrences), lacerations (8 occurrences), lumps (1 occurrence), stab wounds (3 occurrences), gunshot wounds (1 occurrence), and bite wounds (2 occurrences)
Caldas et al. (2012) [19]	Portugal	Observational study design (quantitative)	332 cases of intimate partner violence (69.3% female) with a mean age of 33.7 years were included. Ethnicity not reported	Perioral soft tissues injuries (80.1% (contusion 88.7%, laceration 9.0%, burn 2.3%)), lips injuries (29.5% (contusion 57.1%, laceration 42.9%), gingival and oral mucosa injuries (12.1% (contusion 10.0%, laceration 90.0%) and jaws (1.2%, (contusion 100%)) were common skin injuries. 5.4% participants had perioral soft tissue scars, 6.0% had lip scars and 3.6% had gingival and oral mucosa scars.
Kelkar et al. (2014) [22]	USA	Case Report	One case of a 42-year-old female presented to the emergency department with a history of intimate partner violence (struck by her husband in the right eye with a closed fist). Ethnicity not reported	The case sustained a laceration to her face involving the medial canthus.
Cuculic et al. (2015) [20]	Croatia	Case Report	One case of a 30-year-old woman with multiple skin lesions and a history of intimate partner violence, who was found dead in her fiancés home. Ethnicity not reported	Multiple skin lesions, including three different types of injuries: Contusions, Abrasions and Decubitus ulcers. The injuries involved different body regions: parchment-like circumferential abrasions, covered with scabs, were found on the neck (15 cm 0.5 cm), around the right and left wrist, and left ankle, and all corresponded to ligature marks. An additional abrasion has been documented on the dorsal aspect of the third finger of the right hand. Longitudinal contusions were documented bilaterally along the calves and medial tights, with several minor contusions on the left hand, left upper arm, and dorsum of the left hand. Necrosis was documented on II and III fingers of the left hand, while decubitus ulcers were observed on the left elbow, sacro-coccygeal region, both gluteal regions, and right thigh, left hip, as well as left ankle and right foot and on the back.
Dourado et al. (2015) [21]	Brazil	Observational study design (mixed-method approach)	326 female survivors of intimate partner violence, aged between 18 and 59 years, were included. Skin Color White 10.1% Black 20.2% Dark-skinned 67.2% No information available 2.5%	63.2% women sustained facial injuries (head, neck, and/or face), including lacerations, abrasions, contusions and scars. The qualitative part revealed that visible facial injuries, especially permanent ones, tend to result in feelings of low self-esteem, shame and humiliation in the victim, causing severe psychological distress.
Abedr-Rahman et al. (2017) [17]	Jordan	Observational study design (quantitative)	158 females with a history of intimate partner violence, aged between 18 and 59 years, were included. Ethnicity not reported	Soft tissue injuries accounted for the majority of cases, comprising 96% of the total injuries. Among these, bruises and abrasions were most frequently observed in the upper limbs (27%), followed by the scalp (22%), lower limbs (15%), and black eyes (12%). The neck was affected in 8% of cases, while the nose and back each accounted for 3%. Injuries to the chest and cut wounds at different sites made up 1.7% and 2.3%, respectively. Burns were the least frequent type of injury, making up 1% overall, with scalds, chemical burns, and cigarette burns each accounting for 0.33%. One case was identified as sustaining permanent disability, facial disfiguration due to chemical burns.
Kumala et al. (2020) [23]	Indonesia	Case Report	One case of a 19-year-old female presented to the emergency department with a history of intimate partner violence (doused her face with gasoline and lited a fire by her husband). Ethnicity not reported	Second degree burns to the entire face, up to the ears, neck, chest, both arms and legs. The burns were irregularly shaped, in the form of blackish-brown skin bubbles filled with liquid, accompanied by peeling epidermis.
Sarkar et al. (2020) [24]	Australia	Observational study design (quantitative)	151 cases of intimate partner violence (57.6% female) with a median age of 45 years were included. Ethnicity not reported	Abrasion (18.7%), incised wounds (29.4%), bruises/contusions (18.2%), and laceration (8.9%) were the most common injury types.
Tuncez et al. (2021) [25]	Turkey	Case Reports	Three cases (first 24-year-old woman, second 32-year-old woman, and third 33-year-old woman) presented to the emergency department with a history of intimate partner violence	The first case had large diameter irregular ecchymosis of dark purple and red approximately 30x20 cm area in the posteromedial area and 25x10 cm area in the lateral of right thigh, 2 burned areas of 5x5 and 3x2 cm in the right lumbar region with partially peeled off, irregularly shaped red and green ecchymosis in the area of 6x6 cm in the left upper arm lateral, irregular red, purple and green ecchymotic areas with diameters ranging from 2 to 4 cm on the front of both knees, scar tissue darker then the surrounding tissue in an area of 4x2 cm on the dorsal side of the left hand, scar tissue darker then the surrounding tissue of 0.5 cm width, which is continuous side by side, passing over the proximal phalanges of the 2nd, 3rd, 4th and 5th fingers of the left hand, 4 cm oblique course in right upper arm anterior, 3 cm linear dry abrasion at right forearm lateral and genital organ burns were found. The second case had ecchymoses with blue purple and occasionally yellow green areas in the 50x40 cm starting from the upper left gluteal region and continuing down the thigh posterior, extending to the medial and lateral thigh, 30x15 cm starting from the middle-upper regions of the left cruris lateral to the lateral malleolus, 25x10 cm in the middle anterolateral of the right thigh, 90x40 cm starting from the right scapular region and extending to the right elbow, 10x5 cm in the left scapular region, 40x15 cm posterior to the left upper extremity areas, dark brown healing burned scar areas in the size of 8x4 cm, 5x3 cm in the posterior of the neck and 12x7 cm in the left lateral of the neck, in the middle of the forehead in the size of 2x1 and 5x2 cm sunken than the skin with a mild bleeding scar and an ecchymosis in the right periorbital area. The third case had a wide defect wound under the left knee, open, fragmented fractures in the tibia and fibula, and exposed bone. After the treatments, the patient was operated on and her left leg was amputated below her left knee.
Brady et al. (2023) [11]	USA	Observational study design (quantitative)	133 cases of intimate partner violence (97% female) with a mean age of 34.2±10.3 years were included. Ethnicity Hispanic 41.4% Black 30.1% Non-Hispanic White/Asian 28.6%	Most commonly identified visible signs (bruises, burns, scars, lacerations, wounds) are found on survivors’ neck (80%), face (47%), chin (41%), head (28%), and chest/shoulder area (25%). It was significantly more likely to identify visible signs on White survivors’ chins, neck and torso area. For Hispanic survivors, it was significantly less likely to identify signs of Black survivor’s chins.
Bista et al. (2024) [18]	Nepal	Prospective cohort study	15 female survivors of intimate partner violence, with a mean age of 32.2 years, were included. Ethnicity not reported	All patients (100%) had subconjunctival hemorrhage and ecchymosis. Hyphaema was observed in 13.3% of cases, while lid laceration was present in 6.7% of patients.

**Table 2:** Risk of bias assessment of the observational studies

Author name (year of publication)	Selection (maximum 4*)	Comparability (maximum 2*)	Outcomes (maximum 3*)	Overall (maximum 9*)
Bhandari et al. (2006) [7]	3	1	2	6
Caldas et al. (2012) [19]	3	2	2	7
Dourado et al. (2015) [21]	3	2	2	7
Abedr-Rahman et al. (2017) [17]	3	1	2	6
Sarkar et al. (2020) [24]	3	2	3	8
Brady et al. (2023) [11]	3	1	2	6
Bista et al. (2024) [18]	3	1	2	6

**Table 3:** Risk of bias assessment of the case reports

Author name (year of publication)	Demographic characteristic	Case history	Clinical conditions	Assessment	Treatment procedure	Post-intervention	Unanticipated events	Takeaway lesson	Overall score (Maximum 8)
Kelkar et al. (2014) [22]	Yes	Yes	Yes	Yes	Yes	Unclear	Unclear	Yes	6
Cuculic et al. (2015) [20]	Yes	Yes	Yes	Yes	Unclear	Unclear	Yes	Yes	6
Ria Kumala et al. (2020) [23]	Yes	Yes	Yes	Yes	Yes	Yes	Unclear	Yes	7
Tuncez et al. (2021) [25]	Yes	Yes	Yes	Yes	Yes	Unclear	Unclear	Yes	6

Discussion

This systematic review highlights the dermatological manifestations of domestic abuse across different cultural contexts, emphasizing the significant burden of skin injuries among survivors. The findings demonstrate that bruises, lacerations, burns, and scarring are among the most frequently reported injuries, with variations in presentation influenced by regional and cultural factors. While the types of injuries remain largely consistent globally, certain regions exhibit distinct patterns, such as higher rates of burn-related injuries in South America and Asia or a predominance of facial and neck injuries in North America and Europe. These variations suggest that cultural norms, healthcare accessibility, and reporting practices may influence the recognition and documentation of abuse-related dermatological conditions [26-28].

The role of cultural beliefs and societal attitudes in shaping the response to domestic abuse is evident in the literature [29-31]. In some regions, victims may be reluctant to seek medical care due to stigma, fear of retaliation, or legal barriers [32, 33]. Additionally, healthcare professionals may lack training to recognize dermatological signs of abuse, particularly in cultures where domestic violence is underreported [34-36]. This underscores the need for culturally competent healthcare strategies that integrate dermatological assessments into routine clinical screenings for domestic abuse.

This review has several limitations. First, the included studies varied in methodological rigor, with some relying on self-reported data, which may introduce recall bias. Second, the majority of studies focused on female survivors of intimate partner violence, limiting the generalizability of findings to other populations, such as male victims and children. Third, the lack of standardization in reporting dermatological injuries across studies made it difficult to directly compare findings. Additionally, most of the included studies were observational, limiting causal inferences.

The findings have important implications for practice, policy, and research. Healthcare providers play a crucial role in identifying dermatological signs of abuse and offering appropriate interventions. Training programs should incorporate dermatological manifestations of domestic abuse into medical and nursing curricula, equipping practitioners with the skills to detect and document these injuries accurately. Multidisciplinary collaboration among dermatologists, forensic experts, and mental health professionals is essential for a holistic approach to care. Policymakers should consider integrating routine dermatological screenings into domestic violence intervention programs. Strengthening legal frameworks to protect survivors and improving access to healthcare services can help mitigate the long-term consequences of abuse. Public awareness campaigns should also aim to reduce stigma and encourage early reporting of abuse-related injuries. Future research should focus on longitudinal studies to understand the long-term dermatological consequences of domestic abuse and their psychological impacts. Additionally, there is a need for studies examining the effectiveness of culturally tailored intervention programs. More research is also required on underrepresented populations to develop inclusive and equitable healthcare responses.

## Conclusions

This systematic review underscores the widespread dermatological impacts of domestic abuse, highlighting the need for increased awareness and targeted interventions. While common patterns of injury are observed globally, cultural and regional differences influence how abuse-related dermatological conditions are recognized and managed. Addressing these disparities requires a multifaceted approach, including improved clinical training, policy reforms, and further research into culturally sensitive healthcare responses. By integrating dermatological assessments into domestic violence protocols, healthcare systems can play a pivotal role in early detection and intervention, ultimately improving outcomes for survivors worldwide.

## Appendices

**Table 4:** MEDLINE search strategy Ovid MEDLINE(R) ALL <1946 to February 07, 2025>

S. No.	Search Term / MeSH / Keyword	Result Count
1	exp Domestic Violence/	52,330
2	exp Intimate Partner Violence/	14,429
3	Spouse Abuse/	7,620
4	Battered Women/	2,728
5	Gender-Based Violence/	783
6	Elder Abuse/	3,066
7	family violence.mp.	2,156
8	interpersonal violence.mp.	2,831
9	1 OR 2 OR 3 OR 4 OR 5 OR 6 OR 7 OR 8	62,564
10	Skin Diseases/	64,491
11	Cicatrix/	27,595
12	Dermatology/	22,262
13	Contusions/	5,435
14	Burns, Chemical/ OR Burns/ OR Eye Burns/	57,741
15	"Wounds and Injuries"/	83,626
16	skin trauma.mp.	380
17	scars.mp.	23,597
18	skin injuries.mp.	1,284
19	bruises.mp.	1,680
20	Lacerations/ OR Wounds, Nonpenetrating/	27,882
21	skin lesions.mp.	33,082
22	cutaneous manifestations.mp.	5,664
23	10 OR 11 OR 12 OR 13 OR 14 OR 15 OR 16 OR 17 OR 18 OR 19 OR 20 OR 21 OR 22	326,546
24	Cross-Cultural Comparison/ OR Cultural Diversity/ OR Cultural Characteristics/	57,273
25	Culture/	34,673
26	Ethnicity/	77,415
27	geographic variation.mp.	4,847
28	racial differences.mp.	8,493
29	Stereotyping/	12,528
30	Sociocultural factors.mp.	2,364
31	Customs.mp.	3,151
32	24 OR 25 OR 26 OR 27 OR 28 OR 29 OR 30 OR 31	186,963
33	9 AND 23 AND 32	67

**Table 5:** Embase search strategy Embase <1974 to 2025 February 07>

S. No.	Search Term	Hits
1	domestic violence/	12,691
2	partner violence/	18,697
3	Spouse Abuse.mp.	423
4	battered woman/	3,335
5	gender based violence/	2,009
6	elder abuse/	2,164
7	family violence/	4,314
8	interpersonal violence.mp.	3,343
9	1 or 2 or 3 or 4 or 5 or 6 or 7 or 8	40,037
10	skin disease/	84,734
11	scar/	45,804
12	dermatology/	53,522
13	contusion/	15,826
14	burn/	69,915
15	wound/ or chronic wound/	42,422
16	skin injury/	19,119
17	bruises.mp.	2,538
18	laceration/	16,438
19	skin defect/	72,446
20	cutaneous manifestations.mp. or skin manifestation/	42,443
21	10 or 11 or 12 or 13 or 14 or 15 or 16 or 17 or 18 or 19 or 20	424,430
22	Culture.mp. or cultural anthropology/	1,748,110
23	cultural safety/ or cultural background/ or cultural diversity/ or cultural value/ or cultural identity/	10,496
24	ethnicity/	140,204
25	geographic distribution/ or geographic variation.mp.	189,276
26	race difference/	57,378
27	stereotyping/	3,457
28	social aspect/ or Sociocultural factors.mp. or cultural factor/	152,788
29	Customs.mp.	3,744
30	22 or 23 or 24 or 25 or 26 or 27 or 28 or 29	2,253,260
31	9 and 21 and 30	44

**Table 6:** AMED search strategy AMED (Allied and Complementary Medicine) <1985 to December 2024>

S.No.	Search Term	Hits
1	Domestic Violence/	112
2	Intimate Partner Violence.mp.	58
3	Spouse Abuse.mp.	11
4	Battered Women.mp.	5
5	Gender-Based Violence.mp.	5
6	Elder abuse/	67
7	family violence.mp.	13
8	Violence/ or interpersonal violence.mp.	552
9	1 or 2 or 3 or 4 or 5 or 6 or 7 or 8	735
10	Skin disease/	1,303
11	Cicatrix/	105
12	Dermatology.mp.	134
13	Contusions/	28
14	Burns/	515
15	Burns/	515
16	Wounds/	508
17	skin trauma.mp.	3
18	scars.mp.	85
19	skin injuries.mp.	7
20	bruises.mp.	47
21	Lacerations.mp.	30
22	skin lesions.mp.	126
23	cutaneous manifestations.mp.	8
24	10 or 11 or 12 or 13 or 14 or 15 or 16 or 17 or 18 or 19 or 20 or 21 or 22 or 23	2,717
25	Culture/ or Ethnic groups/	3,309
26	Cross cultural comparison/	345
27	geographic variation.mp.	10
28	racial differences.mp.	68
29	Stereotyping/	63
30	Sociocultural factors.mp. or Socioeconomic factors/	1,339
31	Customs.mp.	44
32	women.mp. or Women/	13,279
33	25 or 26 or 27 or 28 or 29 or 30 or 31 or 32	17,822
34	9 and 24 and 33	1

**Table 7:** CINHAL

S4	S1 AND S2 AND S3	Expanders - Apply equivalent subjects Search modes - Proximity	Interface - EBSCOhost Research Databases Search Screen - Advanced Search Database - CINAHL Complete	69
S3	Culture OR cultural OR Cross-Cultural Comparison OR Cultural Diversity OR Cultural Characteristics OR Ethnicity OR geographic variation OR racial differences OR Stereotyping OR Sociocultural factors OR Customs OR stigma	Expanders - Apply equivalent subjects Search modes - Proximity	Interface - EBSCOhost Research Databases Search Screen - Advanced Search Database - CINAHL Complete	347,535
S2	Skin Diseases OR Cicatrix OR Dermatology OR Contusions OR burns OR ( wounds and injuries ) OR skin trauma OR scars OR skin injuries OR bruises OR Lacerations OR skin lesions	Expanders - Apply equivalent subjects Search modes - Proximity	Interface - EBSCOhost Research Databases Search Screen - Advanced Search Database - CINAHL Complete	132,303
S1	Domestic Violence OR intimate partner violence OR Spouse Abuse OR Battered Women OR Gender-Based Violence OR Elder Abuse OR family violence OR interpersonal violence	Expanders - Apply equivalent subjects Search modes - Proximity	Interface - EBSCOhost Research Databases Search Screen - Advanced Search Database - CINAHL Complete	35,348

Cochrane library

Domestic Violence OR intimate partner violence OR Spouse Abuse OR Battered Women OR Gender-Based Violence OR Elder Abuse OR family violence OR interpersonal violence 3673

Skin Diseases OR Cicatrix OR Dermatology OR Contusions OR burns OR wounds OR injuries OR skin trauma OR scars OR skin injuries OR bruises OR Lacerations OR skin lesions 165773

Culture OR cultural OR Cross-Cultural Comparison OR Cultural Diversity OR Cultural Characteristics OR Ethnicity OR geographic variation OR racial differences OR Stereotyping OR Sociocultural factors OR Customs OR stigma 6125

(Domestic Violence OR intimate partner violence OR Spouse Abuse OR Battered Women OR Gender-Based Violence OR Elder Abuse OR family violence OR interpersonal violence) AND (Skin Diseases OR Cicatrix OR Dermatology OR Contusions OR burns OR wounds OR injuries OR skin trauma OR scars OR skin injuries OR bruises OR Lacerations OR skin lesions) AND (Culture OR cultural OR Cross-Cultural Comparison OR Cultural Diversity OR Cultural Characteristics OR Ethnicity OR geographic variation OR racial differences OR Stereotyping OR Sociocultural factors OR Customs OR stigma) 15

Web of Science

(((((((ALL=(Domestic Violence)) OR ALL=(Intimate Partner Violence)) OR ALL=(Spouse Abuse)) OR ALL=(Battered Women)) OR ALL=(Gender-Based Violence)) OR ALL=(Elder Abuse)) OR ALL=(family violence)) OR ALL=(interpersonal violence) 106,164

((((((((((((ALL=(Skin Diseases)) OR ALL=(Cicatrix)) OR ALL=(Dermatology)) OR ALL=(Contusions)) OR ALL=(Burns)) OR ALL=(Wounds )) OR ALL=(skin trauma)) OR ALL=(scars)) OR ALL=(skin injuries)) OR ALL=(bruises)) OR ALL=(Lacerations)) OR ALL=(skin lesions)) OR ALL=(cutaneous manifestations)))))) 1,377,035

((((((((ALL=(Cross-Cultural Comparison)) OR ALL=(Cultural Diversity)) OR ALL=(Cultural Characteristics)) OR ALL=(Culture)) OR ALL=(Ethnicity)) OR ALL=(geographic variation)) OR ALL=(racial differences)) OR ALL=(Stereotyping)) OR ALL=(Sociocultural factors)))))) 3,397,284

(((((((ALL=(Domestic Violence)) OR ALL=(Intimate Partner Violence)) OR ALL=(Spouse Abuse)) OR ALL=(Battered Women)) OR ALL=(Gender-Based Violence)) OR ALL=(Elder Abuse)) OR ALL=(family violence)) OR ALL=(interpersonal violence) AND ((((((((((((ALL=(Skin Diseases)) OR ALL=(Cicatrix)) OR ALL=(Dermatology)) OR ALL=(Contusions)) OR ALL=(Burns)) OR ALL=(Wounds )) OR ALL=(skin trauma)) OR ALL=(scars)) OR ALL=(skin injuries)) OR ALL=(bruises)) OR ALL=(Lacerations)) OR ALL=(skin lesions)) OR ALL=(cutaneous manifestations)))))) AND ((((((((ALL=(Cross-Cultural Comparison)) OR ALL=(Cultural Diversity)) OR ALL=(Cultural Characteristics)) OR ALL=(Culture)) OR ALL=(Ethnicity)) OR ALL=(geographic variation)) OR ALL=(racial differences)) OR ALL=(Stereotyping)) OR ALL=(Sociocultural factors)))))) 49

Scopus

Domestic Violence OR intimate partner violence OR Spouse Abuse OR Battered Women OR Gender-Based Violence OR Elder Abuse OR family violence OR interpersonal violence 45,019

Skin Diseases OR Cicatrix OR Dermatology OR Contusions OR burns OR wounds OR injuries OR skin trauma OR scars OR skin injuries OR bruises OR Lacerations OR skin lesions 496,678

Culture OR cultural OR Cross-Cultural Comparison OR Cultural Diversity OR Cultural Characteristics OR Ethnicity OR geographic variation OR racial differences OR Stereotyping OR Sociocultural factors OR Customs OR stigma 154,990

(Domestic Violence OR intimate partner violence OR Spouse Abuse OR Battered Women OR Gender-Based Violence OR Elder Abuse OR family violence OR interpersonal violence) AND (Skin Diseases OR Cicatrix OR Dermatology OR Contusions OR burns OR wounds OR injuries OR skin trauma OR scars OR skin injuries OR bruises OR Lacerations OR skin lesions) AND (Culture OR cultural OR Cross-Cultural Comparison OR Cultural Diversity OR Cultural Characteristics OR Ethnicity OR geographic variation OR racial differences OR Stereotyping OR Sociocultural factors OR Customs OR stigma) 61
